# Dysregulated VEGF/VEGFR-2 Signaling and Plexogenic Lesions in the Embryonic Lungs of Chickens Predisposed to Pulmonary Arterial Hypertension

**DOI:** 10.3390/ijms25084489

**Published:** 2024-04-19

**Authors:** Lujie Ye, Rui Liu, Qinghao Li, Chunzhen Zhou, Xun Tan

**Affiliations:** 1 Department of Veterinary Medicine, Zhejiang University, Hangzhou 310058, China; 2 Center for Veterinary Sciences, Zhejiang University, Hangzhou 310058, China; 3 Institute of Preventive Veterinary Sciences, Zhejiang University, Hangzhou 310058, China

**Keywords:** chicken embryo, plexiform lesion, pulmonary arterial hypertension, oxidative stress, HIF-1α, Nrf2, VEGF-A, VEGFR-2

## Abstract

Plexiform lesions are a hallmark of pulmonary arterial hypertension (PAH) in humans and are proposed to stem from dysfunctional angioblasts. Broiler chickens (*Gallus gallus*) are highly susceptible to PAH, with plexiform-like lesions observed in newly hatched individuals. Here, we reported the emergence of plexiform-like lesions in the embryonic lungs of broiler chickens. Lung samples were collected from broiler chickens at embryonic day 20 (E20), hatch, and one-day-old, with PAH-resistant layer chickens as controls. Plexiform lesions consisting of CD133+/vascular endothelial growth factor receptor type-2 (VEGFR-2)+ angioblasts were exclusively observed in broiler embryos and sporadically in layer embryos. Distinct gene profiles of angiogenic factors were observed between the two strains, with impaired VEGF-A/VEGFR-2 signaling correlating with lesion development and reduced arteriogenesis. Pharmaceutical inhibition of VEGFR-2 resulted in enhanced lesion development in layer embryos. Moreover, broiler embryonic lungs displayed increased activation of HIF-1α and nuclear factor erythroid 2-related factor 2 (Nrf2), indicating a hypoxic state. Remarkably, we found a negative correlation between lung Nrf2 activation and VEGF-A and VEGFR-2 expression. In vitro studies indicated that Nrf2 overactivation restricted VEGF signaling in endothelial progenitor cells. The findings from broiler embryos suggest an association between plexiform lesion development and impaired VEGF system due to aberrant activation of Nrf2.

## 1. Introduction

Pulmonary arterial hypertension (PAH) in humans is a disease of the lung vascular system that is characterized by pathologic remodeling of distal arterioles that leads to progressive narrowing of the blood vessels, resulting in a progressive elevation in pulmonary artery pressure and a decrease in distal perfusion [1,2]. The clinical manifestations of PAH may include symptoms such as dyspnea, fatigue, chest discomfort, cyanosis, and lower extremity swelling. At the histological level, severe PAH is marked by the presence of the so-called plexiform lesions, occlusive, glomeruloid-like vascular structures typically occurring distal to branch points of small pulmonary arteries [3,4]. These structures have been identified in individuals across various age groups [3,5], and the earliest occurrence of the lesions has been documented during in utero stages, exemplified by a case involving a 31-week stillborn fetus with premature closure of the ductus arteriosus and lung hypoplasia [6]. Although the role of these lesions in the pathogenesis/progression of PAH is still unclear, patients with plexogenic arteriopathy tend to be unresponsive to vasodilator therapy and have a poor prognosis for survival [7].

It has long been considered that the formation of plexiform lesions is a result of disordered angiogenesis since several angiogenesis-related molecules, including vascular endothelial growth factor (VEGF), VEGF receptor type 2 (VEGFR-2, KDR/Flk-1), angiopoietin (Ang)-1, Ang receptor Tie-2, hepatocyte growth factor (HGF), and transforming growth factor (TGF)-β1, are highly expressed within the lesions [3,8,9]. Consistent with this conception, it has been found that pulmonary artery endothelial cells in adult PAH patients have an apoptosis-resistant and hyperproliferative phenotype in cell culture [10]. A current concept is that plexiform lesions arise from progenitor endothelia cells (EPCs) [11], as is evident by the accumulation of CD133+/VEGFR-2+ progenitor cells in remodeled pulmonary arteries and plexiform lesions [12,13]. However, whether the EPCs in diseased pulmonary arteries originate from bone marrow or from vascular wall-resident EPCs remains controversial [14,15]. On a more basal level, whether and how EPCs contribute to the lesion development remains largely unknown.

Various rodent models of PAH have been established over the years [16]. However, only limited multiple-pathological-insult rat models of PAH have been shown to develop complex plexiform-like lesions, such as monocrotaline (MCT) combined with pneumonectomy [17] or chronic hypoxia [18], or SU5416 (a tyrosine-kinase inhibitor of VEGFR-2) with chronic hypoxia [19] or pneumonectomy [20], providing evidence that severe hypoxia and VEGF/VEGFR-2 signaling dysregulation are required for the development of plexiform lesions. Despite these insights, there is still debate about the relevance of these models in accurately replicating the natural course of plexiform-like lesions and PAH in diseased patients.

While laboratory rodents do not develop plexiform-like lesions without inducing factors, fast-growing broilers are highly susceptible to PAH and can develop plexiform-like lesions spontaneously [21,22,23,24,25]. The lesions in the lung of broiler chickens exhibit anatomical distribution and histological features closely resembling that of human PAH [25,26,27,28,29]. In addition, our group have recently identified CD133+/VEGFR-2+ EPCs in the lesions and proposed a concept that the formation of plexiform lesions is associated with the phenotypical change of EPCs to macrophages [30,31]. Furthermore, studies have shown that the incidence and density of plexiform-like lesions in broilers tend to increase with elevated pulmonary pressure [22,32]. Unexpectedly, our previous studies have shown that the development of plexiform lesions in post-hatch broilers is not associated with any of the major angiogenic pathways [32].

Indeed, several studies, including our own, have documented the presence of plexiform lesions in newly hatched broilers [29,32], suggesting that these lesions may originate during embryonic development and persist post-hatch. This may provide an explanation for the absence of an association between angiogenic factors and plexiform lesion development in post-hatch birds. However, direct evidence of plexiform lesions in chicken embryos is currently unavailable.

Formation of blood vessels during embryonic development is through two major cellular processes: vasculogenesis and angiogenesis [33]. The early development is initiated by the differentiation of the mesodermally derived endothelial precursor cells (“angioblasts” in embryos and “EPCs” in adults) into endothelial cells and coalesce to form a primitive vascular network. New blood vessels are then formed from pre-existing blood vessels either through sprouting or intussusception [34], followed by a process of maturation termed arteriogenesis through the recruitment of mural cells, e.g., pericytes and vascular smooth muscle cells (SMCs) [33]. It is well known that the signaling of VEGF and VEGFR-2 plays an indispensable role in vascular development [35], and that the expression of VEGF is chiefly, positively regulated by transcription factor hypoxia-inducible factor (HIF)-1α under hypoxia [36,37]. However, there is also evidence that chronic hypoxia compromises the angiogenic potential of EPCs [38,39]. Given that oxygen consumption of broiler embryos is higher than that of layers (a strain of chickens bred for egg production known to be genetically resistant to PAH) [40], we hypothesized that broiler embryo can develop plexiform lesions reflecting abnormal VEGF signaling and oxidative stress.

## 2. Results

### 2.1. Plexiform Lesion Incidence and Density in Broilers and Layers

**Figure 1:**
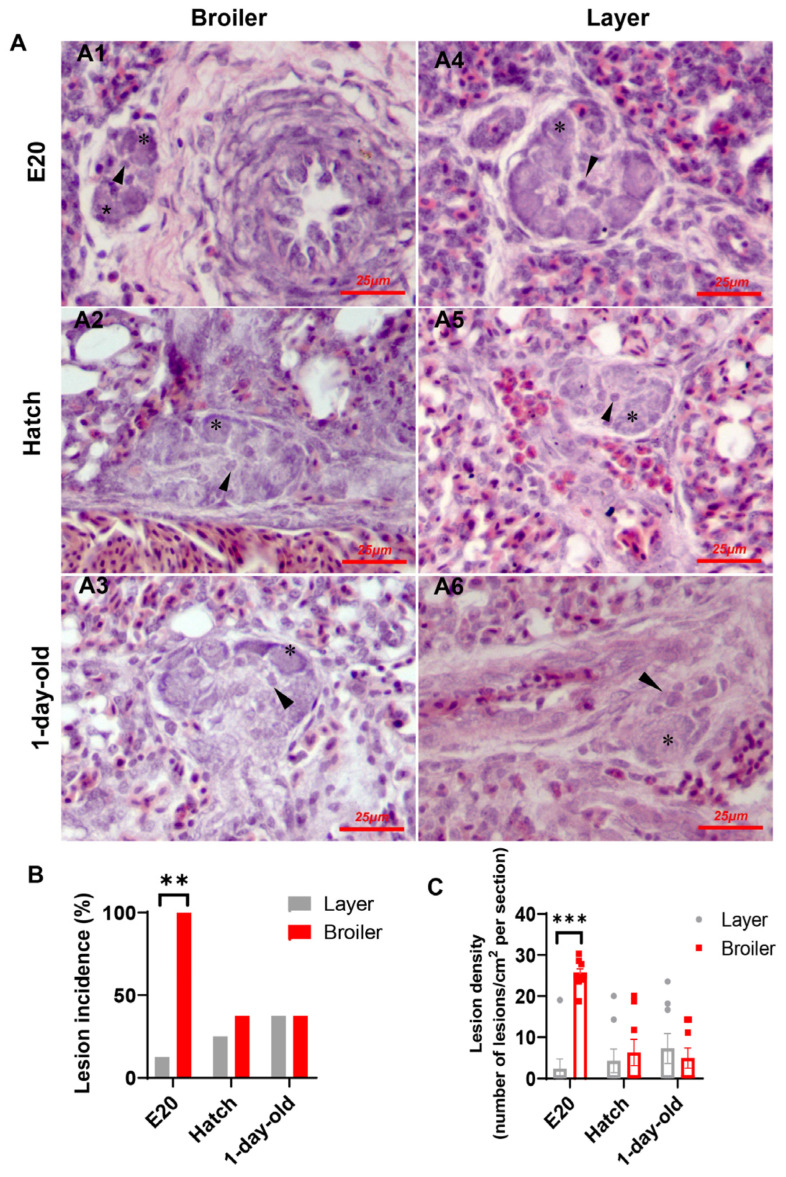
Incidence and density of plexiform lesions in lungs of boilers and layers at different stages of development. (**A**) Histological cross-sections (H&E staining) of lung samples showing morphological characteristics of plexiform lesion in broilers (**A1**–**A3**) and layers (**A4**–**A6**). Plexiform lesions are primarily composed of angioblast-like cells (asterisk) surrounding a core of more differentiated endothelial progenitor cell-like cells (arrowhead). Typically, lesions are located at branching point of artery (**A2**–**A5**) or origin of a supernumerary artery (**A1**,**A6**). (**B**) Plexiform lesion incidence, ** *p* < 0.01. (**C**) Plexiform lesion density. Data are expressed as means ± SEM of 8 birds, *** *p* < 0.001.

Plexiform-like lesions were observed consistently in both broiler and layer strains across all ages sampled, with no apparent morphological differences between the two strains (Figure 1A). The lesions were primarily composed of angioblast-like cells arranged peripherally, with scattered endothelial progenitor-like cells located in the core. The lesions in both chicken strains were located at either arterial branch points or the origin of supernumerary arteries. However, while the lesions were exclusively observed in all lung sections from broiler embryos, only a sporadic occurrence of lesions was observed in the embryonic lungs of layers (Figure 1B). Furthermore, the lesion density, as calculated in terms of lesions observed per square centimeter of the histological section evaluated, was significantly higher in broilers than in layer chicks at E20 (Figure 1C). These findings suggest that broilers are more susceptible to developing plexiform-like lesions during the embryonic development and provide evidence that the lesions may persist into post-hatch life. In addition to plexiform lesions, we frequently observed medial fibromuscular dysplasia (FMD) in the embryonic lungs of broilers, characterized by the formation of a cellular plug obstructing the vessel lumen (Supplementary Figure S1). In contrast, FMD was rarely observed in layer chicks.

### 2.2. Phenotypic Characteristics of Cells in Plexiform Lesions

**Figure 2:**
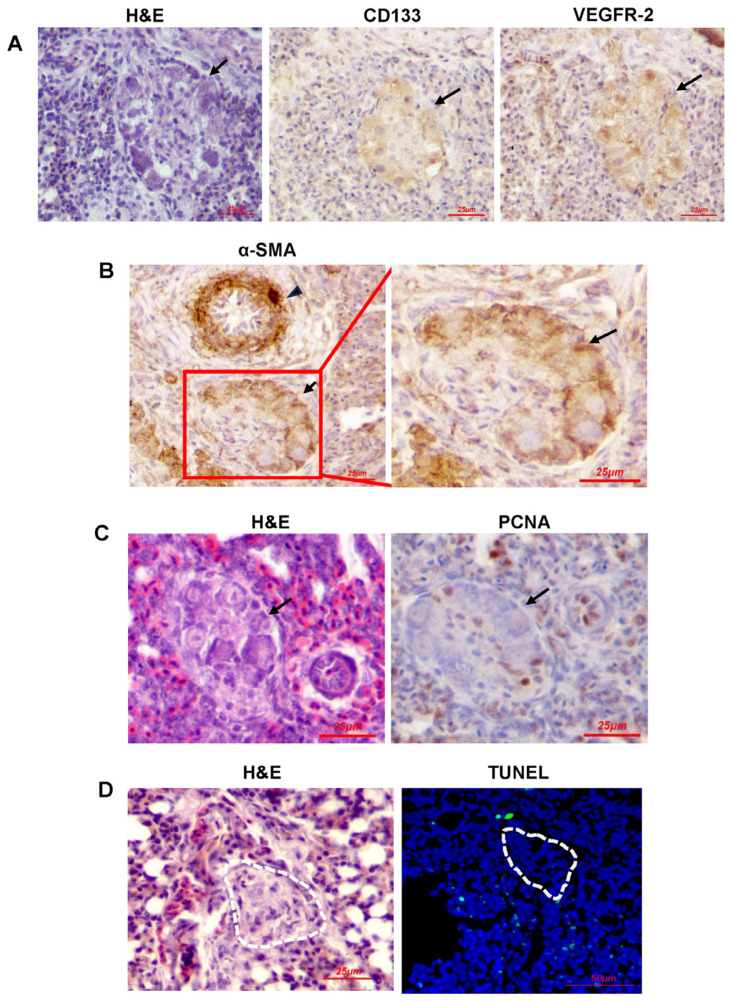
Characterization of angioblast phenotype, proliferation, and apoptosis of cells within plexiform lesions. (**A**) Adjacent sections of lung tissue from a broiler chicken at E20 showing immunohistochemical staining of CD133 and VEGFR-2. Arrow indicates plexiform lesion. (**B**) Lung tissue from broiler chicken at E20 showing immunohistochemical staining of α-smooth muscle actin (α-SMA) of plexiform lesion (arrow) adjacent to pulmonary vessel (arrowhead). (**C**) Lung tissue from a broiler chicken at E20 showing immunohistochemical staining of proliferating cell nuclear antigen (PCNA). Note the weak positive reaction of PCNA in plexiform lesion. (**D**) Lung tissue from a broiler chicken at E20 showing apoptotic cells determined by TUNEL staining (green). Cell nuclei were labeled by DAPI (blue). The dotted lines represent the edge of the lesion. Note the lack of TUNEL-positive cell in plexiform lesion (arrow). For all the experiments, hematoxylin and eosin (H&E) staining was performed to show the histopathologic features of the plexiform lesions. Photographs are from one representative experiment.

To investigate the cellular components of the lesions in our chicken embryo model, we performed immunochemical analysis to detect EPC markers. Our results from broiler embryos, present in Figure 2A, demonstrated that the lesions were primarily composed of CD133+/VEGFR-2+ cells, consistent with the characteristics of angioblasts/EPCs. Intriguingly, these cells were also positive for α-smooth muscle actin (α-SMA) (Figure 2B), indicative of the phenotype of a myofibroblasts. In addition, cells in the lesions exhibited weak or absent immunoreactivity for the proliferation marker PCNA (Figure 2C), suggesting limited proliferative ability. Additionally, almost no apoptotic cells were determined within the lesions by TUNEL assay (Figure 2D).

### 2.3. Broilers Have Decreased Arteriogenesis in Their Lung Compared to Layers

**Figure 3:**
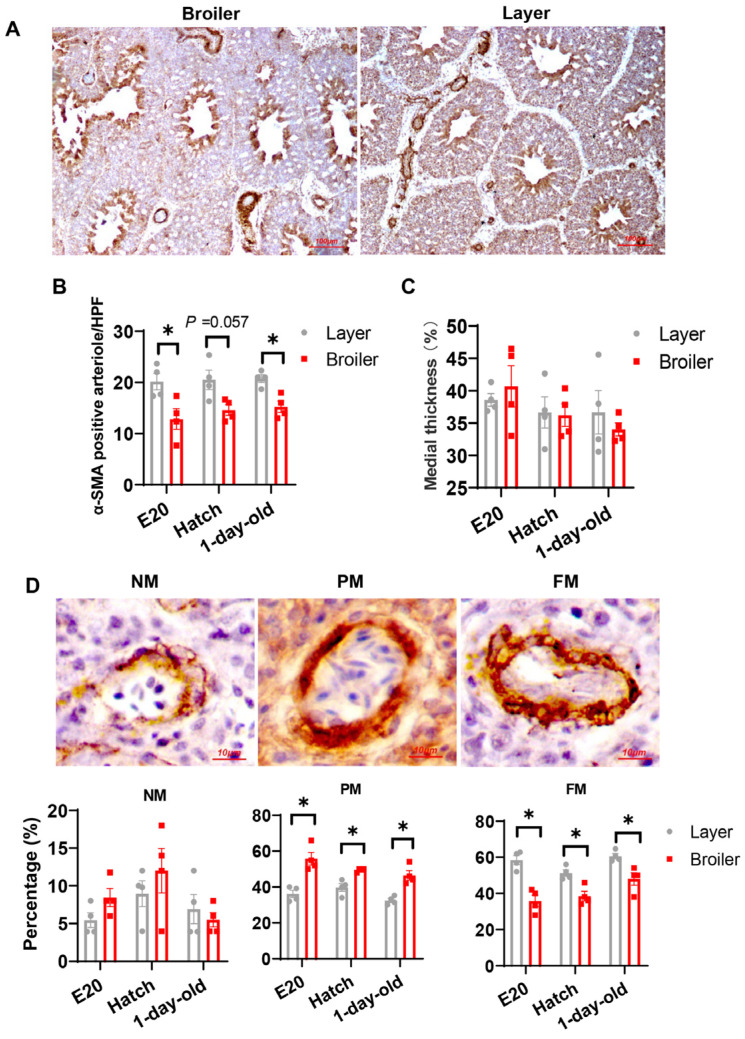
Decreased vasculogenesis in lungs of broilers. (**A**) α-Smooth muscle actin (α-SMA) immunostaining was performed on lung sections to visualize small pulmonary arteries. Representative light microphotographs are shown. (**B**) Pulmonary arterial density. α-SMA-positive vessels were counted under a light microscope. At least three high power fields (HPF) (at ×100 magnification) analyzed in each section. Data are expressed as means ± SEM of 4 birds from each group. * *p* < 0.05. (**C**) Percentage of medial thickness. Data are expressed as means ± SEM of 4 birds from each group. At least four arteries in each section were randomly selected for analysis. (**D**) Muscularization of small pulmonary arteries. Total of 50 α-SMA-positive blood vessels were analyzed in each section to determine percentage of non-muscular (NM), partially muscularized (PM) and fully muscularized (FM) arterioles. Data are expressed as means ± SEM of 4 birds from each group. * *p* < 0.05.

The α-SMA immunostained sections (Figure 3A) were also used to measure arteriole density, percent medial thickness as well as percent muscularization of blood vessels [37,41]. As shown in Figure 3B, there were no age-related trends of arteriole densities within each strain across all sampling ages; however, broilers consistently demonstrated lower arteriole densities than layers. Although no differences in thickness of the medial layer in small pulmonary arteries (20–100 µm) were detected between the strains (Figure 3C), the broilers showed a lower percentage of fully muscularized vessels and a higher percentage of partially muscularized vessels compared to layers (Figure 3D).

### 2.4. Correlations between Lung Angiogenic Factors and Morphometric Parameters Measured

**Figure 4:**
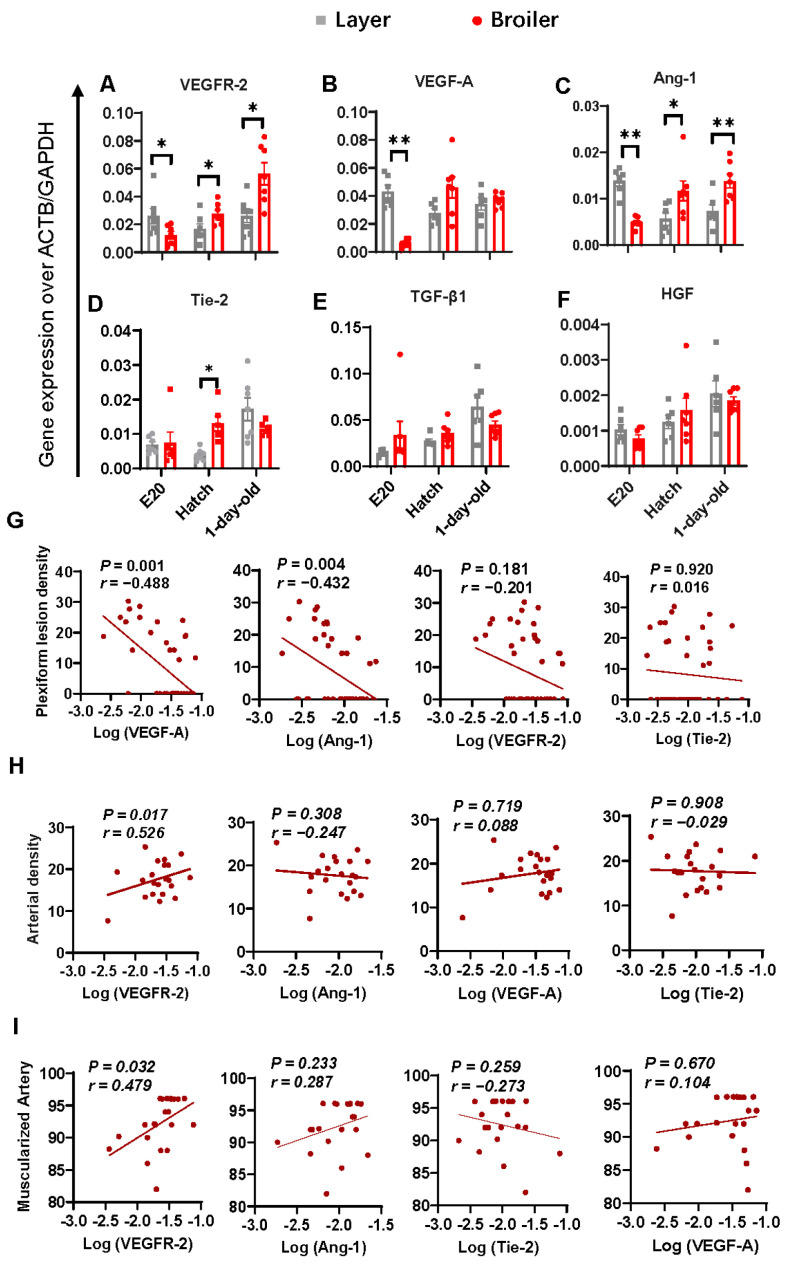
Identification of pro-angiogenic genes related to plexiform lesion formation and arteriologenesis. (**A**–**F**) mRNA levels of lung angiopoietin (Ang)-1 (**A**), vascular endothelial growth factor receptor (VEGFR)-2 (**B**), VEGF-A (**C**), angiopoietin receptor Tie-2 (**D**), transforming growth factor (TGF)-β1 (**E**), and hepatocyte growth factor (HGF) (**F**) were quantified by qPCR. Data are expressed as mean ± SEM of seven broilers and six layers at different stages of development. * *p* < 0.05, ** *p* < 0.01. (**G**–**I**) Partial correlation analysis. The log-transformed mRNA levels of pro-angiogenic genes investigated were plot against plexiform lesion density (**G**), arterial density (**H**), and the percentage of muscularized artery (partially muscularized artery + fully muscularized artery) (**I**) (broiler, *n* = 12; layer, *n* = 12). Correlation coefficient (r) and *p* value are given. A *p*-value < 0.05 indicates significant correlation.

We conducted qPCR analysis to measure the mRNA levels of six angiogenic factors in the lung, including VEGFR-2, VEGF-A, angiopoietin (Ang)-1, angiopoietin receptor Tie-2, transforming growth factor (TGF)-β1 and hepatocyte growth factor (HGF) (Figure 4A–F). Distinct expression patterns of VEGFR-2, VEGF-A, Ang-1 and Tie-2 were found between the broiler and layer strains. To investigate the significance of these factors in relation to the formation of plexiform lesions, partial correlation analysis was performed using the plexiform density as the objective variable after adjustment of age and strain. The factors that were identified to be significantly correlated with the plexiform density, in order of the degree of correlation (co-efficient of standard partial correlation), were VEGF-A and Ang-1, with both factors showing a negative correlation with the plexiform density (Figure 4G). In addition, a positive correlation was found between VEGFR-2 expression and arterial density (Figure 4H), and between VEGFR-2 and vascular muscularization (Figure 4I). No correlation between other angiogenic factors and the morphometric parameters was noted.

### 2.5. Pharmaceutical Inhibition of VEGFR-2 Promotes the Development of Plexiform Lesions in the Embryonic Lung of Layers

**Figure 5:**
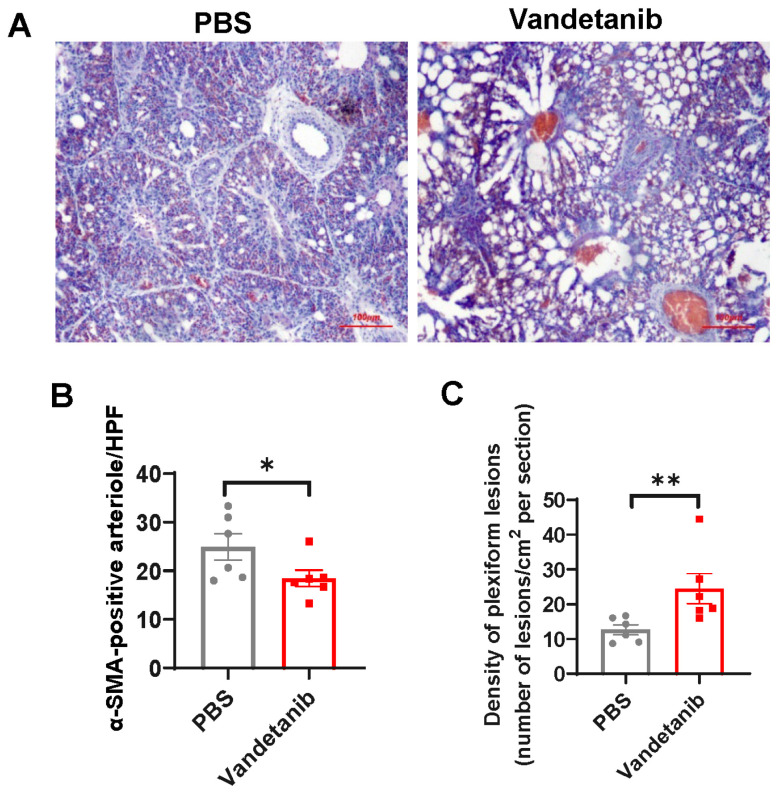
Effect of VEGFR-2 inhibition on alveolarization (**A**), arteriogenesis (**B**), and plexiform lesion formation (**C**). Layer embryonic eggs were treated with VEGFR-2 inhibitor vandetanib or PBS at embryonic day 13 (E13) and lungs were collected at E20. Representative microphotographs are shown. Data are expressed as means ± SEM of 6 birds from each group (vandetanib treatment and PBS treatment). * *p* < 0.05, ** *p* < 0.01.

To verify the role of VEGF-A/VEGFR-2 system in the development of plexiform lesions, we administrated a single dose of vandetanib, a potent inhibitor of the tyrosine kinase activity of VEGFR-2, to layer embryonic eggs at E13. This was conducted because the capillary plexus in the avian lung undergoes rapid expansion from E15 [41], and we wanted to ensure that the treatment was given during this critical developmental period. All embryos survived the treatment. At E20, vandetanib-treated embryos exhibited impaired alveolarization (Figure 5A) and reduced pulmonary arterial density (Figure 5B) compared to the Mock controls, confirming the effectiveness of the inhibitor. As expected, plexiform lesion density in the embryonic lung was significant elevated when VEGF-A/VEGFR-2 pathway was blocked by vandetanib (Figure 5C). We did not evaluate the role of Ang-1 in the development of plexiform lesions.

### 2.6. Nrf2 Is Hyperactivated in Broiler Embryonic Lung and Overexpression of Nrf2 Suppresses VEGF and VEGFR-2 Transcription

**Figure 6:**
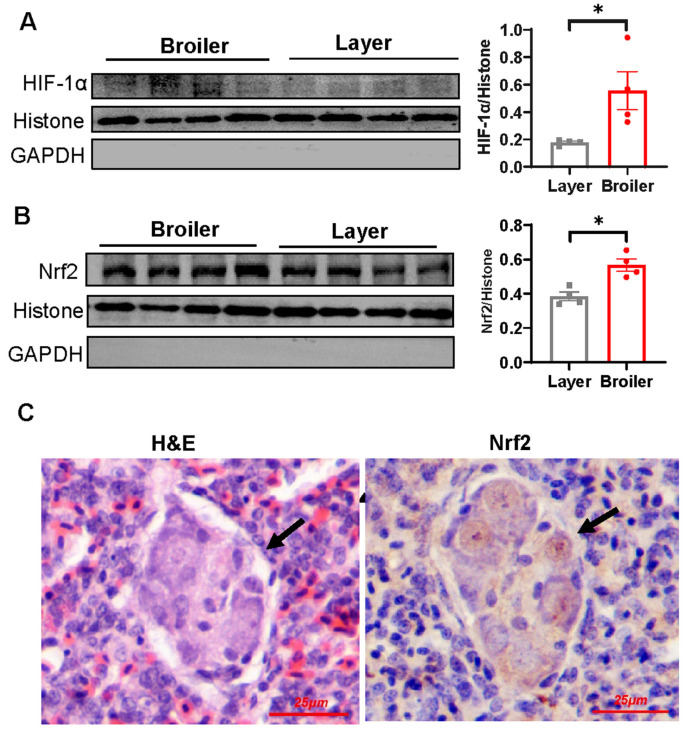
Evaluation of hypoxia response and oxidative stress of broiler embryonic lung. (**A**,**B**) Nuclear protein of lung samples from broiler and layer embryos at E20 were extracted for Western blot analysis using an anti-HIFα (**A**) or anti-Nrf2 (**B**) antibody. Histone and GAPDH are shown as loading control, respectively. Data are expressed as means ± SEM of 4 birds in each group (broiler chickens and layer chickens) and are representative of two independent experiments. * *p* < 0.05. (**C**) Representative image showing immunohistochemical staining of Nrf2 of lung tissue from a broiler at E20. Arrow indicates plexiform lesion.

As our data indicated reduced VEGF-A and VEGFR-2 mRNA levels in the embryonic lung of broilers compared to that of layers, we sought to analyze whether this was due to reduced HIF-1α response. However, Western blot analysis demonstrated an increase of nuclear HIF-1α level in the broiler embryonic lung (Figure 6A). To further understand the molecular mechanisms behind the impaired transcription of VEGF-A and VEGFR-2 in the broiler embryonic lung, we turned our attention to Nrf2, a redox-sensitive transcription factor that is known to regulate the expression of multiple genes involved in cellular stress and antioxidant defense [42,43]. Aberrant activation of Nrf2 has been previously linked to EPC dysfunction and plexiform lesion development [30]. In good agreement with the HIF-1α stabilization and activation, broiler embryonic lungs demonstrated increased nuclear Nrf2 level as compared to that of layers (Figure 6B). Additionally, strong immunostaining for Nrf2 was observed within the lesions (Figure 6C).

**Figure 7:**
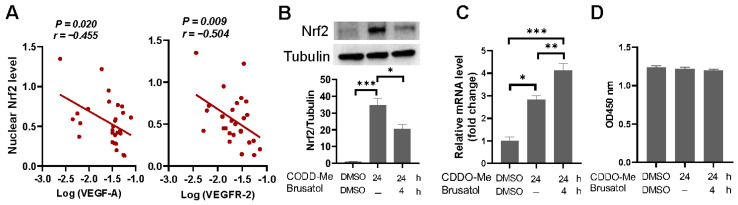
Impact of aberrant Nrf2 pathway activation on VEGF system. (**A**) Association of nuclear Nrf2 levels with VEGF-A and VEGFR-2 mRNA expression. Quantitative Western blot-based lung Nrf2 levels were plotted against log-transformed mRNA levels of VEGF-A (left panel) and VEGFR-2 (right panel) (broiler, *n* = 12; layer, *n*= 12). Correlation coefficient (r) and *p* value are given. A *p*-value < 0.05 indicates significant correlation. (**B**–**D**) Evaluation of Nrf2 hyperactivation on VEGF-A mRNA expression and cell viability in early endothelial progenitor cells (EPCs). Cells exposed to CDDO-me (300 nM) for 24 h with or without the addition of Brusatol (40 nM) to the culture medium 4 h prior to the experimental endpoint and proceeded to immunoblot (**B**), qPCR (**C**) and cell proliferation analyses (**D**). Data are means ± SEM of triplicates and are representative of two independent experiments. * *p* < 0.05, ** *p* < 0.01 and *** *p* < 0.001 (one-way ANOVA with Fisher’s LSD post-hoc test).

We also investigated the nuclear Nrf2 levels in the post-hatch lungs of both broilers and layers. Our analysis did not reveal any statistically significant difference between the two strains, although broilers exhibited comparatively lower Nrf2 levels in their lungs as compared to layers at the age of 1-day-old (Figure S2). Partial correlation analysis indicated that higher nuclear Nrf2 protein levels were associated with reduced levels of VEGF-A and VEGFR-2 mRNA when considering both broilers and layers across all age groups together and after adjusting for age and strain differences (Figure 7A). To confirm that Nrf2 overactivation could downregulate the transcription of these proangiogenic factors, we conducted a dual luciferase reporter assay in HEK-293T cells, which showed that Nrf2 enhanced the VEGF-A promoter activity at low dose while decreased the activity at higher dose (Figure S3). Similar effects of Nrf2 on VEGFR-2 promoter activity were observed (Figure S3).

We then used an early EPC (eEPC) model to investigate the impact of Nrf2 hyperactivation on the VEGF system. We treated the cells with 2-cyano-3,12-dioxooleana-1,9(11)-dien-28-oic acid–methyl ester (CDDO-Me), a potent activator of the Nrf2 signaling pathway, to stabilize intracellular Nrf2. CDDO-Me induced a strong accumulation of intracellular Nrf2 as compared to unstimulated cells (Figure 7B), accompanied by a significant upregulation in VEGF-A mRNA expression (Figure 7C). Notably, the expression of VEGF-A mRNA in CDDO-Me-treated cells was further upregulated when Nrf2 pathway was blocked by Nrf2 inhibitor Brusatol (Figure 7B,C), confirming that excessive activation of Nrf2 restricts VEGF-A expression. In contrast to VEGF-A, VEGFR-2 mRNA level was undetectable in the cultured eEPCs, and CDDO-Me treatment showed only limited effect on the expression of this gene. Thus, we could not definitively conclude the role of Nrf2 in the regulation of VEGFR-2 expression in our eEPC model. In addition, we did not determine significant effects of CDDO-Me or CDDO-Me combined with Brusatol on cell viability (Figure 7D).

In context, our findings suggest a likelihood that aberrant activation of Nrf2 contributes to the development of plexiform lesions by interfering with the VEGF system.

## 3. Discussion

In this study, we report for the first time the presence of plexiform lesions in the embryonic lung of avian species, which appears to persist into post-hatch life. The lesions were found at either arterial branch points or the origin of supernumerary arteries, resembling that observed in human PAH patients [19,44]. Our results support the notion that VEGF-A/VEGFR-2 signaling impairment contributes to the development of plexiform lesions and suggest that aberrant activation of Nrf2 serves as a driving force behind the pathological process.

The cellular origin of plexiform lesions in human PAH remains a conundrum. However, the current concept posits that the development of plexiform lesions is associated with dysfunction in endothelia precursor cells [13,14], which is highlighted by the presence of EPCs within the lesions. In line with these observations, CD133+/VEGFR-2+ angioblasts were found to constitute the predominant cellular component in the plexiform-like structures in our avian embryo model, regardless of the strains. Interestingly, these angioblasts in the lesions also exhibited characteristics reminiscent of myofibroblasts, implying a potential differentiation of angioblasts to myofibroblasts in the progression of lesion development. While further investigations are necessary to validate this hypothesis, there is evidence supporting the involvement of myofibroblast differentiation of EPCs in intimal remodeling in PAH patient [45]. In addition, we found almost no detectable apoptosis in the structures. This concurs with the findings describing the absence of apoptotic cells in the plexiform lesions in the lung of PAH patients [46]. In context, findings from the very-early-stage plexiform-like lesions as observed in our avian embryo model are in consistent with the notion that plexiform lesions originate from angioblasts.

In this work, we present several findings supporting the idea that the impairment in VEGF-A/VEGFR-2 signaling contributes to the development of plexiform lesions, as previously described in the SU5416/hypoxia rat model [19]. Firstly, we observed reduced expression of VEGF-A and VEGFR-2 in the embryonic lung of broilers, which coincided with an increase in the incidence and density of plexiform lesions and reduction in pulmonary arterial density when compared to layers. Furthermore, pharmacological inhibition of VEGFR-2 was sufficient to promote the formation of plexiform lesions in layer embryos, which normally only occur sporadically under normal conditions. Consistent with the findings in infant rats [47], VEGFR-2 inhibition was also associated with decreased pulmonary arterial density and alveolarization in layer embryos. Thus, our results indicate a similar role of VEGF signaling in regulating pulmonary circulation development and the plexiform lesion formation between mammalian and avian species.

Naturally occurring hypoxia is essential for the functional pulmonary vascular system development in the lung [48]. This involves the activation of HIF-1α, which regulates the transcription of a large number of proangiogenic factors including VEGF and VEGFR-2 [49]. However, mounting evidence suggests that pathological HIF-1α activation contributes to vascular lesions in human patients with idiopathic PAH [50] and in experimental PAH [51,52]. Similarly, our findings revealed increased activation of HIF-1α in broiler embryonic lungs as compared to layers, which is not surprising given the higher oxygen consumption rates in broiler embryos [40]. In this regard, broiler embryos could serve as an effective model for investigating the molecular mechanisms underlying dysregulated hypoxia-induced angiogenesis.

Apart from HIF-1α pathway, hypoxia also leads to the stabilization and activation of Nrf2, a master regulator of antioxidant defense [43]. Although accumulating evidence suggests that Nrf2 plays a critical role in normal angiogenic processes [53,54], Nrf2 overactivation has been shown to suppress angiogenesis in a preeclampsia mouse model by decreasing the expression of angiogenic chemokines and cytokines [55]. Similarly, systemic Nrf2 deletion has been shown to augment angiogenesis after hindlimb ischemia [56]. Our previous findings have demonstrated robust Nrf2 activation in plexiform lesions, with evidence suggesting that persistent hyperactivation of Nrf2 promotes lesion development by inducing the differentiation of eEPCs into macrophage lineage, thereby impeding their angiogenic potential [30]. Consistent with the observations in broiler chickens at 4 weeks of age [30], we observed elevated level of Nrf2 protein in broiler embryonic lungs and robust Nrf2 immunoreactivity within the plexiform lesions. Furthermore, we identified a negative correlation between Nrf2 protein level and the expression of angiogenic factors VEGF-A and VEGFR-2 in the lung. Additionally, we observed that hyperactivation of Nrf2 restricted VEGF-A expression in eEPCs. Taken together, our results support the idea that abnormal Nrf2 activation plays a role in the formation of plexiform lesions in our chicken model.

In light of these findings, targeting the Nrf2 pathway could offer potential opportunities for interventions in plexiform lesions. Unfortunately, our attempts to test this hypothesis with broiler embryos were unsuccessful due to the extremely high mortality following pharmacological inhibition of Nrf2 signaling. For future studies, alternative approaches, such as the lung-tissue specific genetic ablation of Nrf2, may be considered.

It appears unlikely that the development of plexiform lesions in the embryonic chicken lung is associated with prolonged and severe haemodynamic stress as observed in human PAH, when taking into account that cardiac output is only directed through avian lungs shortly before hatching [29]. In contrast, we observed a sudden reduction in both the incidence and density of plexiform lesions in our broiler chicken model immediately after hatching. It is more likely that this decline represents a result of rapid regression of these structures during the post-hatch vascular remodeling, as poorly perfused vessels are predisposing to vasculature pruning [57]. Nevertheless, findings from the current study, coupled with previous investigations [29,32], provide evidence that certain plexiform lesions originated during embryonic stages can endure and persist into post-hatch life. The lesions present in post-hatch broilers appear to undergo an age-related remodeling process, transitioning from an early, cellular morphology to a late, less cellular and more fibrotic morphology [25]. Similarly, chronological evolution of plexiform lesions has been implicated in human patients with congenital heart disease [58]. Thus, additional investigations focusing the molecular mechanisms underlying the evolution process of plexogenic arteriopathy in broiler chickens could potentially unveil therapeutic strategies applicable to human arteriopathy.

## 4. Materials and Methods

### 4.1. Animal Ethics

The animal experiments followed the National Guidelines for the Ethical Review of Laboratory Animal Welfare and were reviewed and approved by the Ethics Committee of the Zhejiang University (ZJU20170554).

### 4.2. Embryo and Chick Preparation

Eggs from the breeders of a Hy-Line white layer strain and an Arbor Acres (AA) broiler strain were incubated in standard thermally manipulated conditions in an automatic incubator until hatch. After hatching, the chickens were kept at a container maintained at 37 °C until use. Chicks at different developmental stages including embryonic day 20 (E20), hatch, and 1-day post-hatch were used. Sex determination was not performed.

### 4.3. Vandetanib Inoculation

At E13, the eggs in the incubator were candled to remove unfertilized eggs and dead embryos. Embryonic eggs from the layer lineage were randomly selected for intra-ovo injection. Briefly, a small hole was drilled at the wide end of the egg after cleaning the eggshell with 75% alcohol. Vandetanib (Beyotime Biotechnology, Shanghai, China) diluted in PBS (75 μg/mL) was injected into the air chamber through the hole without passing through the chamber, with a dose equivalent to 15 μg per egg. PBS-injected eggs were used as control. After the inoculations were completed, the hole was sealed with paraffin solution and the eggs were placed back into the same incubator to allow them to continue developing.

### 4.4. Lung Sampling

The embryos were humanely killed by cooling and the chicks killed by cervical dislocation. The whole left lungs were collected and cut in the transverse plane at the major rib indentations (costal sulci). For histological study, one inter-rib division from the middle of each lung was fixed in 4% paraformaldehyde, and the remaining lungs were deep-frozen immediately after collection and stored in liquid nitrogen until use.

### 4.5. Histological and Immunohistochemical Staining

The paraffin-embedded lung samples were serially cut in the transverse plane at 4–5 μm thickness. One slide was stained with haematoxylin and eosin (H&E) for histological analysis. Immunohistochemistry analyses were performed as previously described [30]. Primary antibodies against CD133 (self-prepared), VEGFR-2 (Boster Biotechnology Technology, Wuhan, China), proliferating cell nuclear antigen (PCNA, Abcam, Cambridge, UK), α-SMA (Boster Biological Technology, Wuhan, China), and Nrf2 (Proteintech, Wuhan, China) were used. Signals were developed using 3,3′-diaminobenzidine (DAB). Sections were counterstained with hematoxylin. Images were acquired by using an optical microscope (Nikon, Eclipse Ci, Tokyo, Japan).

### 4.6. TUNEL Assay

TUNEL assay was performed using the TUNEL BrightGreen Apoptosis Detection Kit (Vazyme, Nanjing, China). Sections were counterstained with 4′,6-diamidino-2-phenylindole (DAPI, Beyotime Technology, Shanghai, China) and examined under a fluorescence microscope (Eclipse Ti, Nikon, Japan).

### 4.7. Quantitative Real-Time RT–PCR (qPCR)

Total RNA was extracted from frozen lung tissue using MolPure^®^ TRIeasy™ Plus Total RNA Kit (Yeasen Biotechnology, Shanghai, China). For cDNA synthesis, 2 μg of total RNA was reverse-transcribed into cDNA using TOROIVD^®^ qRT Master Mix (Toroivd, Shanghai, China) according to the supplier’s instruction. For real-time PCR, reactions were performed on the Roche LightCycler 480 II system (Roche Diagnostics GmbH, Mannheim, Germany) using SYBR Green PCR Master Mix Plus (Vazyme, Nanjing, China), according to the manufacturer’s instructions. Amplification of ACTB and GAPDH mRNA was performed on each sample as a control for normalization. The relative expression of the target genes was also corrected to *ACTB* and *GAPDH* using 2^−ΔCT^ method. The primer sets used were described previously [32].

### 4.8. Western Blot Analysis

Nuclear extracts of the lung samples were prepared by using a Nuclear and Cytoplasmic Protein Extraction Kit (Beyotime Technology, Shanghai, China). Western blot analysis was performed using antibodies against Nrf2 (1:1000; cat# 16396-1-AP, Proteintech, Wuhan, China), HIF-1α (1:1000; cat# sc-13515, Santa Cruz Biotechnology, Santa Cruz, CA, USA), GAPDH (1:2000; cat# FD 0063, Fude Biological Technology, Hangzhou, China), Histone H3 (1:2000; cat# sc-517576, Santa Cruz Biotechnology, CA, USA) and Tubulin (1:2000; cat# 30302E20, Yeasen, Shanghai, China) and horseradish peroxidase (HRP)-conjugated secondary antibody as described previously [30]. The levels of Nrf2 and HIF-1α were analysed by scanning densitometry using ImageJ software (version 1.53c) from the NIH (Bethesda, MD, USA) and were normalised to Histone H3.

### 4.9. Luciferase Assay

The pEGFP-chNrf2 plasmid was prepared as previously descried [30]. A predicted regulatory region containing a 5′-flanking sequence of VEGF-A (from −729 to −1486) as well as VEGFR-2 (from −176 to −980) was cloned into a pGL3-basic vector (Promega, Madison, WI, USA) containing a firefly luciferase reporter gene. For Luciferase assay, HEK293T cells were co-transfected with 1.5 μg of expression plasmid encoding chicken Nrf2 (chNrf2), 500 ng of pGL3 and 100 ng of pRL-TK using the Lipo8000 (Beyotime Technology, Shanghai, China), along with the appropriate amount of empty vectors. After 72 h of transfection and stimulation, luciferase activities were analyzed with the Dual-Luciferase Assay kit (Vazyme, Nanjing, China) and the relative luciferase activities were calculated as the ratio of firefly to Renilla luciferase activity. Luciferase activities were measured in triplicate.

### 4.10. Ex Vivo Expansion of Endothelial Progenitor Cells and Treatments

Ex vivo expansion of early endothelial progenitor cells (eEPCs) was performed exactly as described in our previous work. Briefly, mononuclear cell fraction of the peripheral blood (PBMC) from 4-week-old healthy broiler chickens was cultured in Endothelial cell growth medium (EGM)-2 (Lonza, Walkersvil, MD, USA) containing 2% fetal bovine serum (FBS), 100 U/mL penicillin, and 100 μg/mL streptomycin at 39 °C in 5% CO_2_. Non-adherent cells were removed after 48 h. On day 6 of culture, cells were passaged using 0.25% trypsin/EDTA (Invitrogen), plated on rat tail type 1 collagen-coated 6-well plates at 1  ×  10^7^ cells/well, and treated with 2-cyano-3,12-dioxooleana-1,9(11)-dien-28-oic acid–methyl ester (CDDO-Me) (Merck Ltd., Beijing, China) at 300 nM for 24 h with or without the addition of Brusatol (Merck Ltd., Beijing, China) at 40 nM to the culture medium 20 h after CDDO-Me exposure.

### 4.11. Cell Viability Assay

Cell viability was determined by using Cell Counting Kit-8 (CCK-8, FDbio Science, Hangzhou, China) according to the manufacturer’ instructions on a plate reader at 450 nm.

### 4.12. Statistical Analysis

Plexiform lesion incidences were defined as [(number of lung sections with plexiform lesion)/(number of lung sections examined) × 100], and comparison between strains within a sampling age was performed using Fisher’s exact test. Plexiform lesion density was expressed as the number of lesions/cm^2^ per section, as described previously [32]. Arterial density in each section was calculated as the number of α-SMA-positive blood vessels per high-power filed (at ×100 magnification). Percent medial thickness of α-SMA-positive blood vessels (<100 μm diameter) and the percent muscularization of vessels were calculated as previously described [59,60]. Difference between groups were analyzed by using Student’s *t*-test or non-parametric Mann–Whitney *U*-test, as appropriate, unless otherwise stated. Correlations were determined by using the partial correlation test following logarithmic transformation of the relative amounts of the genes investigated. A *p* value < 0.05 was considered significant. The statistical analysis was performed using SPSS software (version 22; IBM Corp., Armonk, NY, USA).

## 5. Conclusions

In summary, our study reveals that plexiform lesions can develop spontaneously in the lungs of avian embryos, with broiler chickens being more susceptible to the lesion development. These structures appear to persist into post-hatch life and are associated with impaired VEGF/VEGFR-2 signaling. Additionally, our findings suggest that Nrf2 hyperactivation may play a role in the formation of these lesions. Given the striking similarity between broiler embryonic lung and human PAH lung in terms of hypoxia and loss of pulmonary arterioles, shared mechanisms may exist between this avian model and human contexts in the initiation and progression of plexiogenic arteriopathy.

## Data Availability

The datasets used and/or analyzed during the current study are available from the corresponding author on reasonable request.

## Supplementary Materials

The following supporting information can be downloaded at: https://www.mdpi.com/article/10.3390/ijms25084489/s1.
