# Supplementary material for: Dysregulated VEGF/VEGFR-2 Signaling and Plexogenic Lesions in the Embryonic Lungs of Chickens Predisposed to Pulmonary Arterial Hypertension
Source: Int J Mol Sci. 2024 Apr 19;25(8):4489. doi: 10.3390/ijms25084489 (PMC11049811; doi:10.3390/ijms25084489)
Supplement: Supplementary file 1 [file ijms-25-04489-s001.zip › Figure S1.pdf]

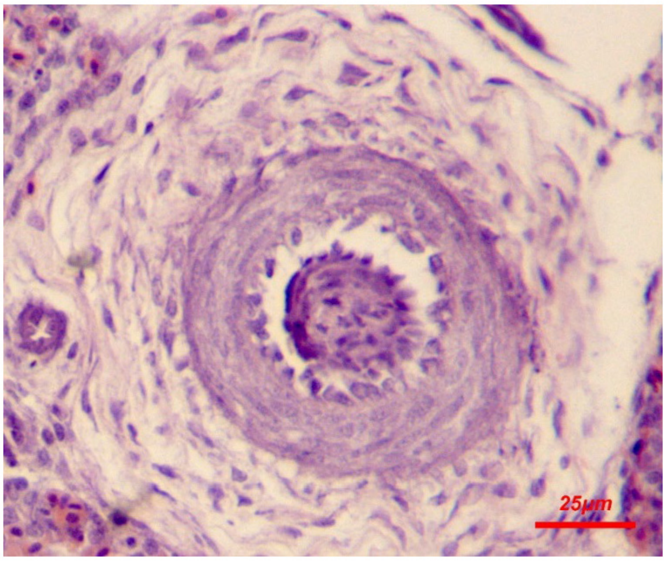

Figure S1. Hematoxylin and eosin-stained lung preparation from a broiler chicken at E20 showing medial fibromuscular dysplasia (FMD).
