# Supplementary material for: Dysregulated VEGF/VEGFR-2 Signaling and Plexogenic Lesions in the Embryonic Lungs of Chickens Predisposed to Pulmonary Arterial Hypertension
Source: Int J Mol Sci. 2024 Apr 19;25(8):4489. doi: 10.3390/ijms25084489 (PMC11049811; doi:10.3390/ijms25084489)
Supplement: Supplementary file 1 [file ijms-25-04489-s001.zip › Figure S2.pdf]

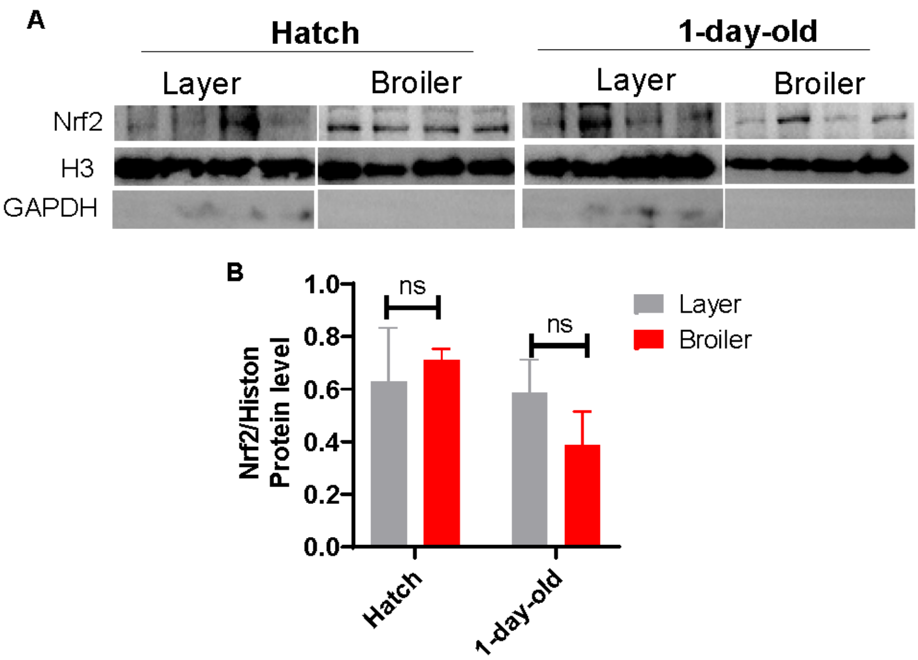

Figure S2. Nuclear Nrf2 levels in the lungs of broilers and layers at hatch and 1-day old. (A) The nuclear fraction of whole lung tissue was subjected to Western blot analysis using an anti-Nrf2 antibody, with Histone H3 and GAPDH serving as loading controls. Representative immunoblots are provided. (B) Quantitative analysis of Nrf2. Data are expressed as means ± SEM of 4 birds in each group. ns: no significant difference.
