# Supplementary material for: Dysregulated VEGF/VEGFR-2 Signaling and Plexogenic Lesions in the Embryonic Lungs of Chickens Predisposed to Pulmonary Arterial Hypertension
Source: Int J Mol Sci. 2024 Apr 19;25(8):4489. doi: 10.3390/ijms25084489 (PMC11049811; doi:10.3390/ijms25084489)
Supplement: Supplementary file 1 [file ijms-25-04489-s001.zip › Figure S3.pdf]

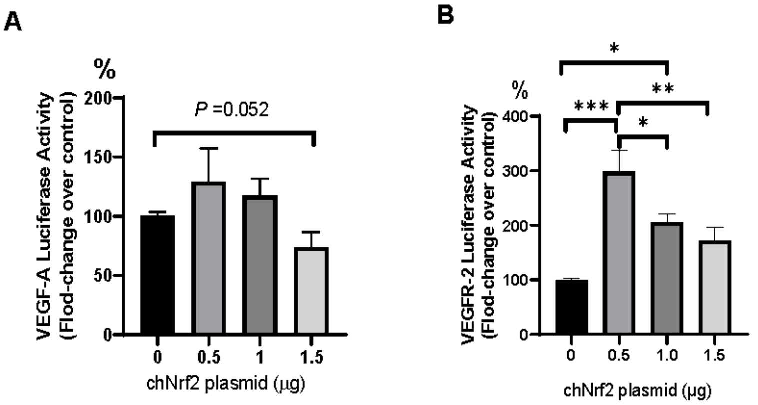

Figure S3. Dual luciferase reporter assay for determination of the response of VEGF-A (A) and VEGFR-2 (B) to Nrf2. HEK-293T cells were co-transfected with the chNrf2 expression vector pCMV-EGFP-Nrf2 (denoted in μg of DNA per well of a 12-well plate) and the plasmid constructs containing a firefly luciferase gene under the control of the VEGF-A or VEGFR-2 promoter along with the pRL-TK Renilla. Empty pCMV-EGFP-flag was used as control. Cells were maintained within a humidified 5% CO2 atmosphere for 72 h before the measurement of luciferase activity. Data are means ± SEM of triplicates and are representative of two independent experiments. * $P < 0.05$, ** $P < 0.01$ and *** $P < 0.001$ (one-way ANOVA with Fisher's LSD post-hoc test).
